# MRI Guided Focused Ultrasound Thalamotomy for Moderate-to-Severe Tremor in Parkinson's Disease

**DOI:** 10.1155/2015/219149

**Published:** 2015-09-02

**Authors:** Ilana Schlesinger, Ayelet Eran, Alon Sinai, Ilana Erikh, Maria Nassar, Dorith Goldsher, Menashe Zaaroor

**Affiliations:** ^1^Department of Neurology, Rambam Health Care Campus, Haifa, Israel; ^2^Technion Faculty of Medicine, Haifa, Israel; ^3^Department of Radiology, Rambam Health Care Campus, Haifa, Israel; ^4^Department of Neurosurgery, Rambam Health Care Campus, Haifa, Israel

## Abstract

*Background*. Thalamotomy is effective in alleviating tremor in Parkinson's disease (PD). *Methods*. Seven PD patients, mean age 59.4 ± 9.8 years (range, 46–74) with a mean disease duration of 5.4 ± 2.8 years (range, 2–10) suffering from severe refractory tremor, underwent ventral intermediate nucleus thalamotomy using MRI guided focused ultrasound (MRgFUS), an innovative technology that enables noninvasive surgery. *Results*. Tremor stopped in the contralateral upper extremity in all patients immediately following treatment. Total UPDRS decreased from 37.4 ± 12.2 to 18.8 ± 11.1 (*p* = 0.007) and PDQ-39 decreased from 42.3 ± 16.4 to 21.6 ± 10.8 (*p* = 0.008) following MRgFUS. These effects were sustained (mean follow-up 7.3 months). Adverse events during MRgFUS included headache (*n* = 3), dizziness (*n* = 2), vertigo (*n* = 4), and lip paresthesia (*n* = 1) and following MRgFUS were hypogeusia (*n* = 1), unsteady feeling when walking (*n* = 1, resolved), and disturbance when walking tandem (*n* = 1, resolved). *Conclusions*. Thalamotomy using MRgFUS is safe and effective in PD patients. Large randomized studies are needed to assess prolonged efficacy and safety.

## 1. Background

Tremor is the most common symptom of Parkinson's disease (PD), observed in about 50% of patients at the time of diagnosis. The tremor is usually a rest tremor but postural/kinetic tremor and reemergent tremor may occur [1]. The tremor interferes with daily living activities and causes social embarrassment and isolation. Current medications primarily target bradykinesia and rigidity with unpredictable responsiveness of tremor [2].

Surgical treatment options are also available for patients with debilitating tremors. Deep brain stimulation (DBS) to the ventral intermediate thalamic nucleus (VIM), globus pallidus internus, and subthalamic nucleus are beneficial in improving PD tremors [3] but their drawbacks are high costs and high complication rates [4].

MRI guided focused ultrasound (MRgFUS) is a new technology that enables noninvasive intracranial focal ablation within the central nervous system, with promising preliminary results [5–7]. Lesions are generated by gradual focal heating with the lesion's location and temperature controlled and monitored by MRI. During the initial reversible low temperature focal heating, patients are observed for transient positive and adverse clinical effects prior to irreversible high temperature ablation. The treatment's impact on the tremor is monitored clinically during the procedure.

We report our experience with thalamotomy using MRgFUS in PD patients with medication resistant tremor.

## 2. Methods

### 2.1. Patients

Seven PD patients (6 males and 1 female) with severe refractory tremor underwent thalamotomy, using ExAblate (Table 1). The diagnosis of idiopathic PD was made according to the UK brain bank criteria. Severe refractory tremor was defined as a disabling tremor despite ample treatment trials with anticholinergic and dopaminergic medication. Severity of tremor was measured by the unified Parkinson's disease rating scale (UPDRS-Part III). A score of 4 on item 20 of the UPDRS was defined as a severe tremor. Disability was defined as interference of tremor in at least two daily living activities. These patients had no contraindications for the procedure including but not limited to significant cognitive decline, current anticoagulant therapy, brain tumors or vascular malformation, significant unstable medical conditions, and contraindications for MR.

### 2.2. Thalamotomy Using MRgFUS

Patients underwent MRI scans using a GE 3-Tesla system and a 3D CT scan. Initial target coordinates of the ventral intermediate nucleus (VIM) were at 25% of the AC-PC distance anterior to the PC and 14 mm lateral to the AC-PC line. VIM thalamotomy was performed contralateral to the more disabling side and according to the preference of the patient.

On the day of the procedure, the patient's head was shaven completely. A stereotactic frame (CRW Integra Plainsboro, NJ) was fixed to the skull and an elastic silicone diaphragm affixed. The patient's head was attached to the focused ultrasound 1024 US transducers helmet. A T2 weighted MRI scan was performed for planning reassessment.

MRgFUS sonications were performed using a 3-Tesla MRI (GE) and a focused ultrasound system (ExAblate Neuro, Insightec).

Treatment included a gradual increase in total energy either by an increased intensity or by longer sonication durations. Sonications were stopped when adequate control of tremor was achieved, with the temperature reaching no more than 59°C.

### 2.3. Statistical Analysis

Pre- and postprocedure total UPDRS scores, scores on items 20 and 21, and Parkinson's disease questionnaire (PDQ39) were compared using a paired *t*-test and were considered significantly different for *p* < 0.05.

## 3. Results

Seven PD patients underwent unilateral MRgFUS VIM thalamotomy, contralateral to the more severe tremulous side. Total average time in the MRI was 250.7 minutes. Total sonication time was 161.4 minutes. All patients were right handed, with tremor more prominent on the right side in four of the patients. The mean age (±SD) of the patients was 59.4 ± 9.8 years (range, 46 to 74) with mean disease duration of 5.4 ± 2.8 years (range, 2 to 10) (Table 1). Follow-up was 3–12 months (mean 8.0 ± 4.1 months).

Tremor was abolished immediately after the procedure in all patients. One patient experienced relief of lower extremity tremor and rigidity as well. Reemergence of short lasting, mild tremor was reported in three patients, one week (*n* = 1), one month (*n* = 1), and half a year (*n* = 1) after the procedure.

The mean UPDRS at baseline was 37.4 ± 12.2 points and decreased to a mean of 18.8 ± 11.1 points one week after the sonications (*p* = 0.007). Item 20 of the UPDRS (rest tremor in the treated side) was improved from a mean baseline score of 2.7 ± 1.1 to a mean score of 0.0 ± 0.0 (*p* < 0.001) and item 21 of the UPDRS (action tremor in the treated side) improved from a mean baseline score of 3.0 ± 1.0 to a mean score of 0.0 ± 0.0 (*p* < 0.001) one week after the procedure. PDQ39 decreased from 42.3 ± 16.4 to 21.6 ± 10.8 (*p* = 0.008) one week after the procedure. This improvement persisted in patients that came for follow-up 3 months after procedure. The minimental state examination was 30 in all patients before and after the procedure. The clinical assessment of the examiner and patients changed from severe disability to no functional disability immediately following the procedure and during follow-up.

Adverse events which occurred during the sonications included headache (*n* = 3), dizziness (*n* = 2), vertigo (*n* = 4), and lip paresthesia (*n* = 1, resolved after target was repositioned 1 mm anteriorly). Adverse events which lasted after the procedure included hypogeusia (*n* = 1), subjective unsteady feeling when walking (*n* = 1, resolved), and disturbance when walking tandem (*n* = 1, resolved at 2-month follow-up).

The MRgFUS resulted in close to a spherical lesion in the planned target with an average volume of 231 ± 64 mm^3^ one day after the procedural scan. The lesion was surrounded by mild edema. Lesion size and surrounding edema slightly increased on the 1-week postprocedural scan to an average of 276 ± 79 mm^3^. In five out of the seven patients with 2-3-month postprocedural scans, lesions markedly decreased in size with an average volume of 25 ± 15 mm^3^.

## 4. Discussion

We report seven PD patients with medication resistant tremor who underwent unilateral VIM thalamotomy using MRgFUS. Tremor was abolished in all patients immediately after the procedure and most side effects were mild and transient. The desired effect was sustained over time. Patients reported improvement in quality of life immediately after the procedure which has continued until present time.

VIM thalamotomy in PD patients with medication resistant tremor has long been known to be effective [8]. In the 1980s, it was considered the treatment of choice in medication resistant PD patients [8] and it has been reported as effective in approximately 80–90% of patients. In 1987, Benabid et al. [9] discovered that stimulation of the VIM thalamus ameliorates tremor and opened a new era of deep brain stimulation to relieve tremor. Patients with PD were first treated with thalamic stimulation to relieve tremor but rigidity and akinesia were not treated effectively by thalamic stimulation. Therefore, globus pallidus internus and subthalamic stimulations are currently the preferred targets in PD patients.

There is a wide range of adverse events related to the surgical procedures, the implanted hardware, and the stimulation [10]. These major adverse effects deter many patients from undergoing invasive surgery.

MRgFUS for the treatment of PD was first reported by Magara et al. [11] in eight patients later expanded to thirteen patients. Nine patients suffered from tremor dominant PD and four from the akinetic form. Eleven patients received levodopa and two discontinued levodopa years before the MRgFUS treatment. However, all had levodopa resistant symptoms. Unilateral pallidothalamic tractotomy was performed using MRgFUS. The first four patients were treated with a single sonication at the peak temperature with a 7.6% reduction of mean total UPDRS. The next nine patients were treated with repeated sonications at peak temperature 4-5 times with a reduction of mean total UPDRS of 60.9%. Posttreatment medications were reduced in five out of the thirteen patients (38%).

Our tremor dominant PD patients were selected for this treatment if they had medication refractory tremor and chose not to undergo DBS. We chose to treat our patients with conventional VIM thalamotomy as opposed to tractotomy chosen by Magara et al. [11] The patients underwent sonication with peak energy given once or twice according to the effect on the tremor. None of our patients received peak energy more than twice, since the achieved effect was deemed satisfactory.

The main advantage of the new MRI guided high intensity focused ultrasound technology allows us to perform VIM thalamotomy with great precision without invasive surgery, through an intact skull. The thermal guidance provided by MR thermal imaging during the procedure, in real time, enables repositioning of the target if there is no clear response of tremor or if adverse effects occur before a nonreversible lesion is created. Thus, in principle, permanent long-term serious side effects can be avoided. Furthermore, the main drawbacks of invasive surgery, implanted hardware, and the need for repeated stimulation adjustment are not applicable here.

The limitations of this report are the small number of patients and the short follow-up period. In addition, both the patients and the physicians knew that treatment was provided. Therefore, the placebo effect and bias of the examining physician cannot be ruled out.

Our initial small cohort of PD patients showed promising results with MRgFUS VIM thalamotomy. Larger studies with sham controls and longer follow-up periods should establish this innovative technique as an efficacious and safe noninvasive and nonpharmacological approach for the beneficial treatment of medication resistant moderate-to-severe tremor in PD.

## Figures and Tables

**Table 1:** Patients characteristics of Parkinson's disease patients that underwent focused ultrasound thalamotomy.

	Patient						
	1	2	3	4	5	6	7
Age, y	61	66	65	51	74	53	46
Sex	Male	Male	Male	Male	Female	Male	Male
Disease duration, y	10	7	7	2	4	5	2.5
Medications	Levodopa Rasagiline Pramipexole	Levodopa Rasagiline	Levodopa Entacapone	Rasagiline Biperiden	Rasagiline Pramipexole Amantadine	Rasagiline Pramipexole	Rasagiline Biperiden
Dominant hand	Right	Right	Right	Right	Right	Right	Right
Treated hand	Right	Right	Right	Left	Right	Left	Right
Postprocedure follow-up, months	12	12	12	8	6	3	3
